# De Novo Assembly and Characterization of Venom Gland Transcriptome for *Rhabdophis lateralis*

**DOI:** 10.3390/toxins18040167

**Published:** 2026-03-30

**Authors:** Jiahao Chen, Qin Liu, Songwen Tan, Peng Guo, Lianming Du

**Affiliations:** 1 Antibiotics Innovation and Resistance Control Key Laboratory of Sichuan Province, Institute for Advanced Study, Chengdu University, Chengdu 610106, China; cjh9057@163.com; 2 Faculty of Agriculture, Forestry and Food Engineering, Yibin University, Yibin 644000, China; ybutsw@163.com; 3 College of Life Science, Sichuan Normal University, Chengdu 610101, China; chimliu@sicnu.edu.cn

**Keywords:** snake venom, *Rhabdophis lateralis*, toxin, transcriptome, phylogenetics

## Abstract

*Rhabdophis lateralis* is a snake species within the family Natricidae, which is widely distributed across mainland China, Russia, and Korea. Although this species was once thought to be non-venomous, there are quite a few cases demonstrating its bite could be fatal. In this study, we performed de novo assembly and analysis of the transcriptome data from the Duvernoy’s gland of *R. lateralis*, aiming to characterize its venom transcriptome and reveal the molecular basis of its toxicity. Among 6196 annotated transcripts, 77 were identified as potential toxin transcripts belonging to 26 toxin families. The most highly expressed toxin family was the SVMP family, accounting for 51.10% of the total toxin expression. The other notable toxins included cysteine-rich secretory proteins (CRISPs, 22.36%), c-type lectins (CTLs and snaclecs, 12.13%), and three-finger toxins (3Ftxs, 6.36%). Phylogenetic analyses indicated that SVMPs, CRISPs, and three-finger toxins (3FTxs) are evolutionarily conserved within Colubridae, whereas CTLs likely arose through convergent evolution. All identified SVMPs were classified as P-III type, with one sequence displaying a unique deletion distinct from conventional truncation patterns. The predominantly expressed CTLs are more likely to combine into dimers, exerting coagulation activity. This study provides an insight into the toxin gene expression in the Duvernoy’s gland of *R. lateralis*, which will benefit future research into the ecological and pharmacological significance of toxins in the genus *Rhabdophis*.

## 1. Introduction

Snakes have undergone extensive evolutionary diversification. Some are classified as front-fanged snakes based on tooth position, such as the Elapidae and Viperidae family; they are typically highly venomous and most venom studies have focused on these species. However, exclusively focusing on these front-fanged species in venom studies may introduce bias, particularly in investigations of venom gene family origins, adaptive evolution, and venom diversification [1]. Researchers have consistently underrepresented rear-fanged snakes, including Colubridae, Homalopsidae, and Lamprophiidae, in modern toxinology [2,3]. These rear-fanged species share some toxin families with front-fanged lineages, including three-finger toxins, metalloproteinases, and phospholipases [1,4,5]. However, their venom composition, delivery system, and functional adaptability have evolved in distinct ways [6]. Because Duvernoy’s glands lack storage sacs and muscle pumps, these snakes do not rely on specialized injection structures. Instead, venom enters prey wounds passively through grooved fangs [7,8,9,10,11,12].

In the family Colubridae (Natricinae), the genus *Rhabdophis* includes both venomous and non-venomous species, reflecting a distinctive venom ecology. The two species with reported envenomation cases are the red-necked keelback (*Rhabdophis subminiatus*) and the tiger keelback (*Rhabdophis tigrinus*). Historically, *R. tigrinus* was divivded into three subspecies—*R. t. tigrinus*, *R. t. lateralis*, and *R. t. formosanus*—before *R. lateralis* was elevated to full species status [13]. In this rear-fanged species, the venom glands are located near the maxilla and are not directly connected to the rear fangs by a duct; venom release depends on bite pressure [14]. Despite the limited venom delivery efficiency, envenomation in Guangxi and Fujian, China, induce hemorrhagic symptoms [15]. Due to taxonomic revisions, historical medical records routinely attributed these bites to *R. tigrinus*. This species possesses two potential toxin-secreting glands: the nuchal gland located behind the head, and the Duvernoy’s gland, which is typical gland of rear-fanged snakes. For a long time, the capacity of these snakes for endogenous toxin synthesis has been debated. The nuchal glands are primarily understood to function by sequestering toxins from prey items, rather than through de novo synthesis. Studies have demonstrated that the nuchal glands contain *bufadienolides* sequestered from dietary sources, such as toads (*Bufonidae*) and certain fireflies (*Lampyrinae*) [16]. Its secretion usually leads to mild neurological damage, such as temporary blindness. Moreover, owing to this species’ relatively low aggressiveness, it has long been regarded as non-venomous in traditional knowledge. This misunderstanding may lead to serious consequences. Victims are often unaware of envenomation at the time of the bite, resulting in delayed medical intervention. In Japan, bites are frequently reported and typically cause coagulopathy. Severe cases may progress to disseminated intravascular coagulation (DIC), and fatalities have been reported [17,18,19].

In this study, we present the first systematic analysis of the Duvernoy’s gland transcriptome of *R. lateralis* and identify its potential toxin components. Toxin composition and expression levels were compared, and major toxin families were structurally characterized via sequence alignments. We also perform phylogenetic comparisons of SVMPs, CRISPs, CTLs, and 3FTxs across snake families to gain insight into the toxin repertoire and evolutionary history of *R. lateralis*.

## 2. Results and Discussion

### 2.1. Transcriptome Assembly and Functional Annotation

Sequencing completed across three samples generated an average of 58,168,780 clean reads and 8,676,564,582 clean bases. The data showed high quality, characterized by average Q20 and Q30 scores of 99.32% and 97.45%, and a GC content of 45.19% (Table 1). Following assembly and redundancy removal, an average of 336,299 contigs were obtained. CDS prediction identified 23,372 sequences with an average length of 1608 bp (Table 2).

**Figure 1:**
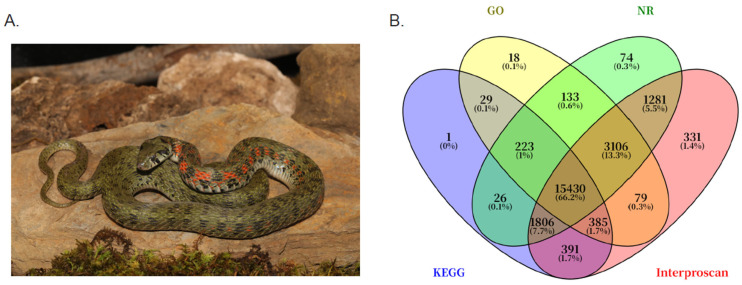
Photograph of *Rhabdophis lateralis* and functional annotation overview. (**A**) Photograph of an adult *R. lateralis* by Tan. (**B**) Venn diagram of the annotated genes across the four databases: NR, GO, KEGG, Interproscan.

Predicted CDSs were translated into protein sequences and aligned against five public databases for functional annotation, including Gene Ontology (GO), Kyoto Encyclopedia of Genes and Genomes (KEGG), NCBI’s non-redundant protein database (NCBI nr) and InterProScan. A total of 23,313 unigenes were annotated across these databases. Venn analysis revealed that 15,430 sequences were commonly annotated by all databases (Figure 1B).

**Figure 2:**
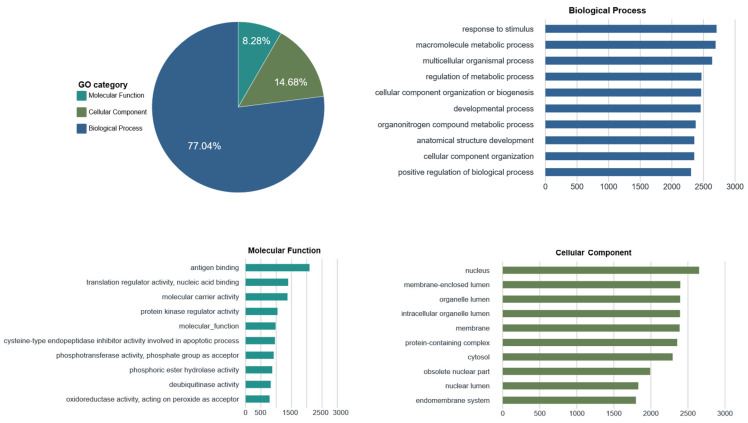
Gene Ontology (GO) functional classification. The pie chart illustrates the distribution of the three main categories: biological process, cellular component, and molecular function. The bar charts display the top 10 most abundant GO terms within each category.

**Figure 3:**
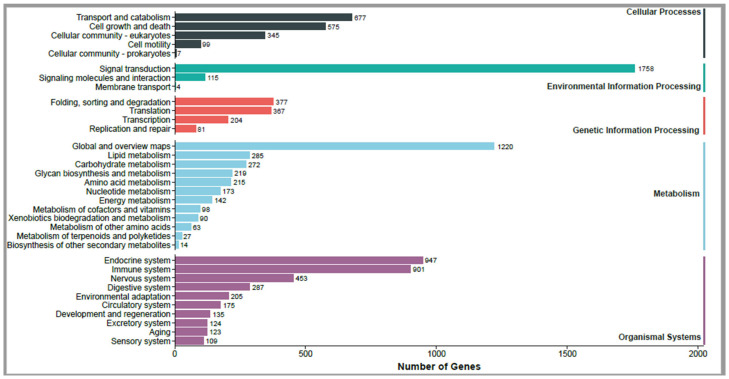
KEGG pathway classification. The x-axis displays the number of genes annotated to each pathway. The left y-axis lists specific kegg pathways, while the right y-axis indicates their corresponding secondary functional categories.

The sequences were classified into three categories based on GO annotation (Figure 2). Biological process (BP) accounted for the majority (77.04%), followed by cellular component (CC, 14.68%) and molecular function (MF, 8.28%). The “response to stimulus” (GO:0050896) category was the most abundant biological process, whereas “antigen binding” (GO:0003823) and “translation regulator activity” (GO:0045182) were the top enriched molecular function terms. Biological functions of the Duvernoy’s gland transcripts were characterized via KEGG pathway analysis (Figure 3). “Signal transduction” (1758 genes) dominated the environmental information processing category, suggesting active intercellular communication, while “global and overview maps” (1220 genes) was the most abundant metabolism pathway. The “endocrine system” and “immune system” pathways were highly represented in the organismal systems category, and “transport and catabolism” (677 genes) dominated cellular processes.

### 2.2. Identification of Potential Toxin Genes

**Figure 4:**
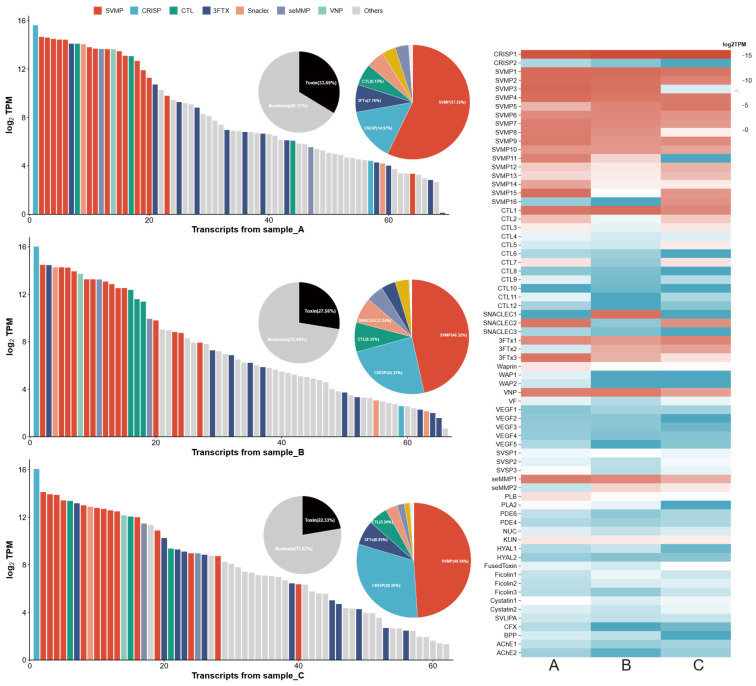
Toxin gene expression profiles and composition in the Duvernoy’s glands of three *R. lateralis* individuals. The bar chart displays the expression levels of individual toxin transcripts, colored by toxin family; the y-axis represents normalized expression values (log2tpm). The black-and-white pie chart indicates the proportion of toxin-encoding transcripts (black) versus non-toxin transcripts (gray) in the total transcriptome. The colored pie chart shows the relative abundance of each toxin family within the toxins. The heatmap illustrates the expression patterns of toxin genes across three samples, where deeper red color indicates higher expression levels.

Assembly and annotation of Duvernoy’s gland transcriptomes from three *R. lateralis* individuals yielded an average of 4004 removed chimeric transcripts per sample. The final consensus assembly comprised 6196 annotated transcripts, including 77 potential toxin sequences spanning 26 families (Figure 4). SVMP was the most highly expressed toxin family; the average proportion of expression in the three samples was 51.10% (TPM range = 10.19–25,768.83, average = 9209.09, *n* = 16), followed by cysteine-rich secretory protein (CRISP, 22.36%, TPM range = 21.33–68,255.82, average = 30,900.05, *n* = 2), c-type lectin (CTL, 6.75%, TPM range = 1.09–22,578.87, average = 2717.11, *n* = 12), three-finger toxin (3Ftx, 6.36%, TPM range = 67.67–10,617.72, average = 5862.69, *n* = 3), snake venom C-type lectins (SNACLEC, 5.38%, TPM range = 1.00–19,950.90, *n* = 3), viper natriuretic peptide (VNP, 3.73%, TPM range = 4569.70–13,584.45, average = 10,298.72, *n* = 1), sea anemone matrix metalloproteinase (seMMP, 3.27%, TPM range = 46.75–12,993.89, average = 4520.12, *n* = 2), and other toxins (1.05%). These minor components included Kunitz-type inhibitor (KUN, TPM range = 438.61–547.01, average = 495.99, *n* = 1), phospholipase B (PLB, TPM range = 225.07–692.72, average = 412.20, *n* = 1), waprin (WAP, TPM range = 1.00–584.16, average = 142.46, *n* = 3), snake venom serine protease (SVSP, TPM range = 29.99–244.13, average = 137.99, *n* = 3), cystatin (TPM range = 44.68–279.67, average = 133.77, *n* = 2), and ficolin (TPM range = 9.71–147.54, average = 60.68, *n* = 3). The predominance of SVMPs, CRISPs, 3FTxs, and CTLs aligns with the typical venom profile of rear-fanged snakes [19].

### 2.3. Sequence and Phylogenetic Analysis of Major Toxins

Life-threatening coagulopathy is frequently documented following *R. tigrinus* envenomation [20]. These severe outcomes are attributed to SVMPs in *R. tigrinus* Duvernoy’s gland secretions [21]. Consistently, abundant SVMP transcripts were identified in *R. lateralis*, which was previously regarded as conspecific with *R. tigrinus*.

**Figure 5:**
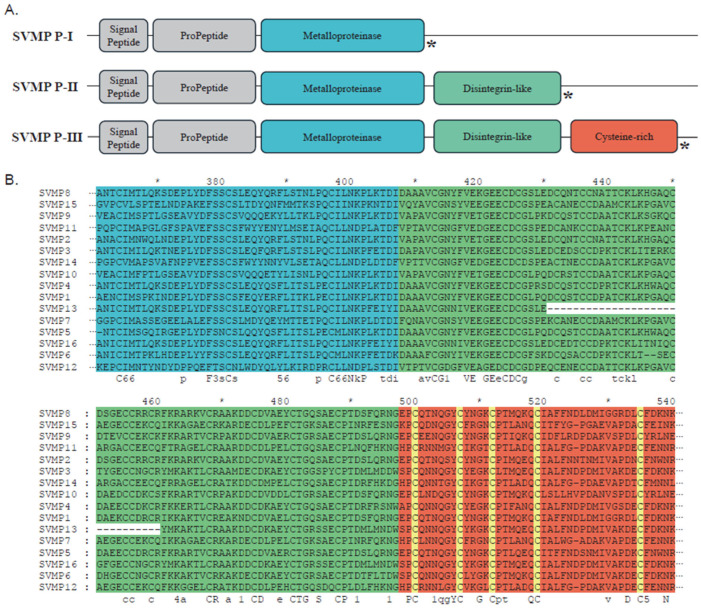
**Main** domain architecture and sequence analysis of snake venom metalloproteinases (SVMPs) in *R. lateralis.* (**A**) Schematic representation of the three SVMP classes (P-I, P-II, and P-III). The asterisk indicates a stop codon. (**B**) Main amino acid sequences of SVMPs identified in *R. lateralis*. Background colors correspond to the domain structures shown in Figure 5A. Cysteine residues are highlighted in yellow. The asterisk denotes every 10 amino acids.

**Figure 6:**
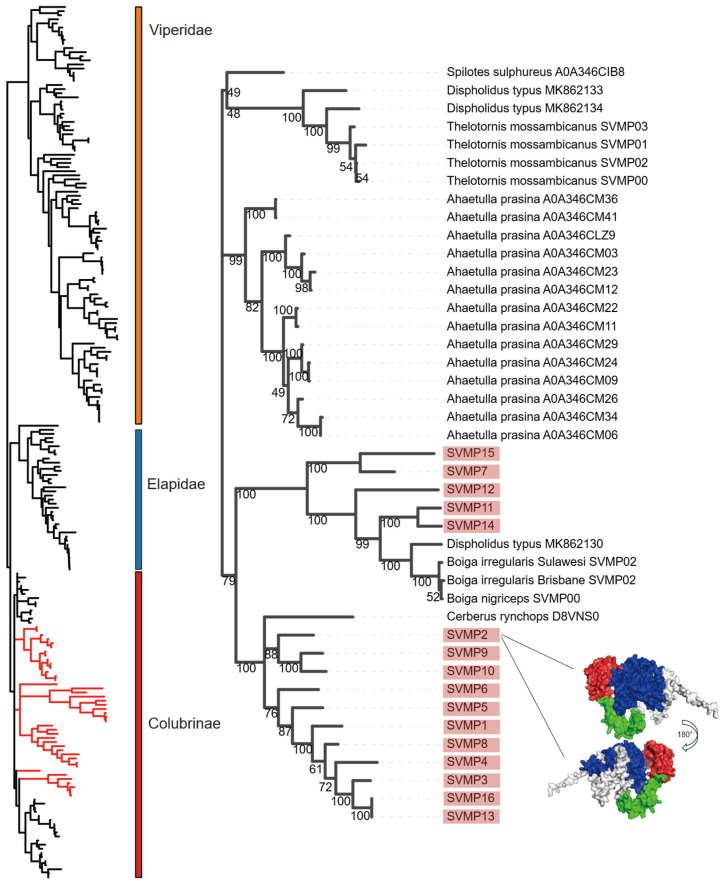
Phylogenetic analysis of snake venom metalloproteinases (SVMPs). **Left**: The overall topology of the phylogenetic tree. The red branch indicates the clade expanded in the right panel. **Right**: Detailed phylogeny of the Colubridae lineage. *R. lateralis* sequences are highlighted with red shading. The sequences used for protein 3D structure prediction have been marked with straight lines; colors indicate domain locations; blue represents the disintegrin-like domain; green represents the peptidase domain; red represents the cys-rich domain. Taxonomic families are distinguished by color blocks: Viperidae (yellow), Elapidae (blue), and Colubridae (red).

The SVMP family has long been recognized as a major component of snake venoms, and its presence is not limited to the Viperidae, Elapidae, and other conventionally venomous snakes. It is also significant in the Colubridae [22,23]. The P-III class represents the archetypal SVMP structure, comprising metalloproteinase, disintegrin, and cysteine-rich domains (Figure 5A). The other classes are derived through domain loss: the P-II type lacks the cysteine-rich domain, while the P-I type consists solely of the metalloproteinase domain [24]. Furthermore, P-III SVMPs comprise two distinct subtypes: P-III-tD, truncated within the disintegrin-like domain, and P-III-tC, truncated within the cysteine-rich domain [25,26]. In both instances, premature translation stop occurs upon entry into the corresponding domain due to the presence of a termination codon. Sixteen SVMP transcript variants were identified across the three *R. lateralis* samples, all classified as P-III SVMPs (Figure 5B). SVMP3 and SVMP11 showed low expression in sample C, whereas SVMP16 was expressed at low levels in samples A and B. All SVMP sequences contained the conserved HEXXHXXGXXH metalloproteinase catalytic motif and retained intact functional domains [27]. However, SVMP13 exhibited a distinct internal deletion in the disintegrin-like domain. Unlike the P-III-tD and P-III-tC subtypes, this deletion was not a truncation but a specific loss of a cysteine-rich region, resulting in eight fewer cysteine residues compared to other SVMPs. This structural variation may reflect the dynamic genetic differentiation mechanisms described by Xie et al. [28]. The phylogenetic tree comprises 213 sequences, predominantly representing the families Viperidae, Elapidae, and Colubridae. While *R. lateralis* sequences clustered within the family Colubridae (Figure 6), the SVMPs formed two distinct monophyletic groups. One clade clustered closely with *Cerberus rynchops* and the other was associated with the genera *Boiga* and *Dispholidus*. These findings suggest that SVMPs in *R. lateralis* exhibit a relatively conserved evolutionary pattern within the Colubridae family.

**Figure 7:**
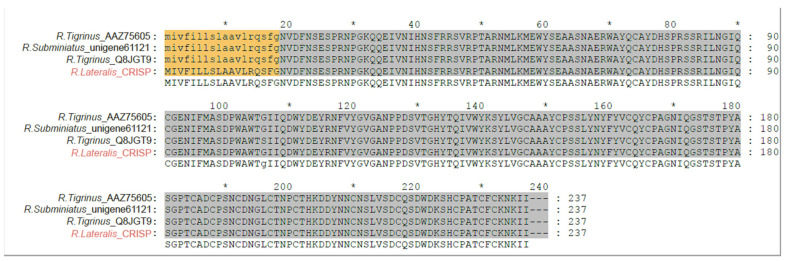
Multiple sequence alignment of cysteine-rich secretory proteins (CRISPs) from three *Rhabdophis* species. The alignment compares amino acid sequences of CRISPs from *R. lateralis*, *R. subminiatus*, and *R. tigrinus*. Signal peptides are highlighted in yellow, and functional domains are shaded in gray. The asterisk denotes every 10 amino acids, and red text represents CRISP detected in this study.

**Figure 8:**
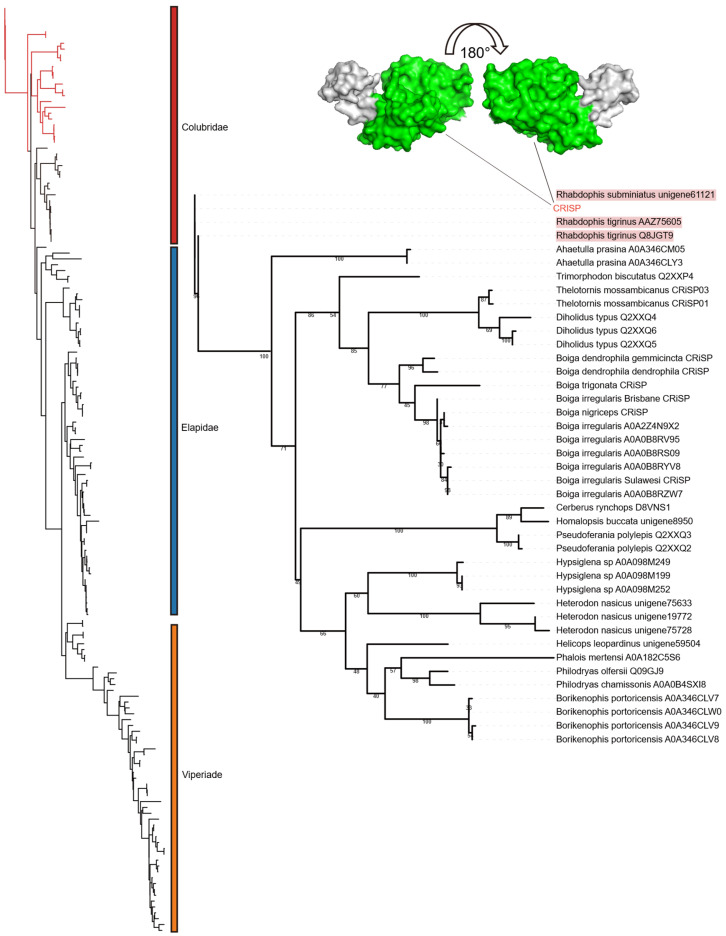
Phylogenetic analysis of cysteine-rich secretory proteins (CRISPs). **Left**: The overall topology of the phylogenetic tree. The red branch indicates the clade expanded in the right panel. **Right**: Detailed phylogeny of the Colubridae lineage. CRISPs identified in this study are indicated in red font, while previously reported *Rhabdophis* CRISP sequences are highlighted with a red background. The sequences used for protein structure prediction have been marked with straight lines; the green in the structure represents the functional domain. Taxonomic families are distinguished by color blocks: Viperidae (yellow), Elapidae (blue), and Colubridae (red).

Although cysteine-rich secretory proteins (CRISPs) are recognized as major components of many snake venoms, their pathological roles remain elusive [29,30]. Experimental studies have shown that CRISPs can inhibit ion channels and exhibit neurotoxicity effects [31,32,33,34]. However, neurotoxic symptoms are typically absent in clinical reports of envenomation by this species [17], suggesting that these proteins may act as adjuncts to the primary coagulation toxins. Nevertheless, the importance of CRISPs should not be overlooked due to their high expression levels. In this study, only a single CRISP transcript was identified, but it was extremely abundant, with an average TPM of 61,790 across the three samples. Moreover, previous studies have shown that CRISPs in rear-fanged snakes display low genetic diversity but relatively high expression levels, suggesting that the functional contribution of this toxin family may rely less on toxin diversification than on high expression to achieve biological efficacy [35]. The identified CRISP sequences were identical to previously reported *Rhabdophis* CRISPs (Figure 7). The phylogenetic tree included 159 sequences, primarily from Viperidae, Elapidae, and Colubridae. Consistent with the SVMP results, the *R. lateralis* CRISP clustered with Colubridae (Figure 8). However, *R. subminiatus* and *R. tigrinus* form a distinct clade in the phylogenetic tree, suggesting that this lineage evolved independently following ancestral divergence [30].

**Figure 9:**
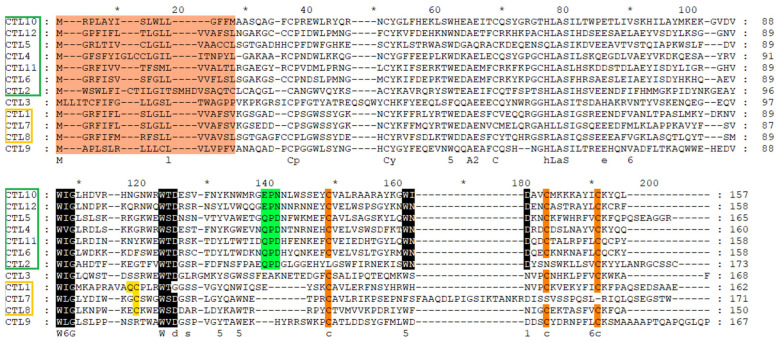
Multiple sequence alignment of C-type lectins (CTLs) identified in *R. lateralis*. In the sequence ID panel, green boxes indicate CTLs retaining the ancestral motif, while yellow boxes denote potential dimer-forming sequences. In the alignment, the ancestral motifs are highlighted in green, residues distinguishing α and β chains are marked in yellow, and conserved cysteines are shaded in orange. The asterisk denotes every 10 amino acids.

**Figure 10:**
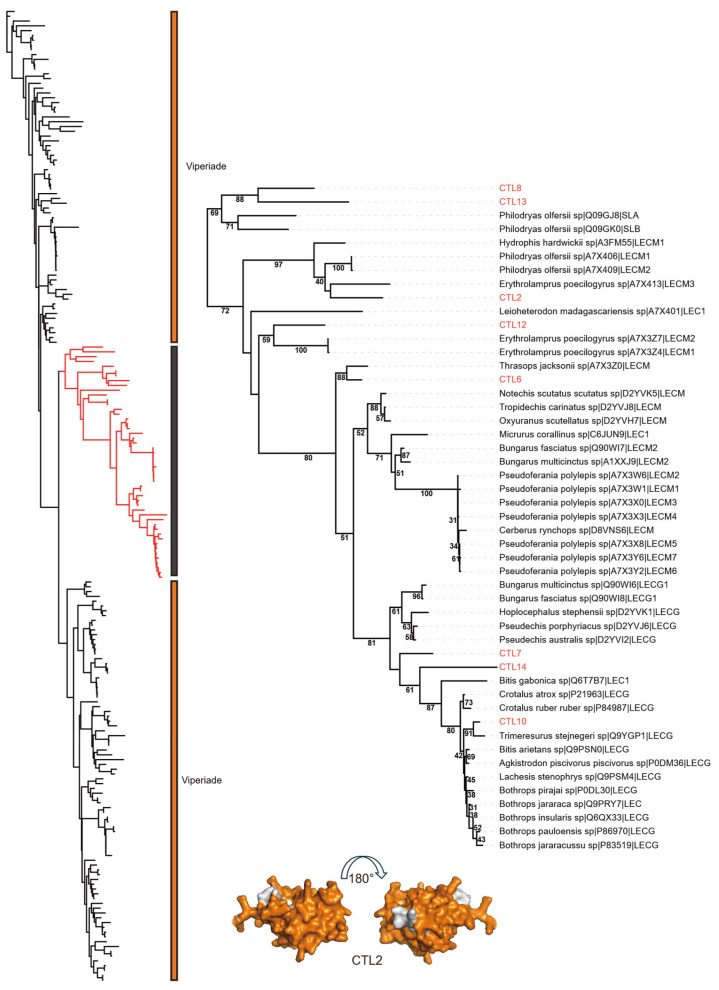
Phylogenetic analysis of C-type lectin (CTLs). Left: The overall topology of the phylogenetic tree. The red branch indicates the clade expanded in the right panel. Right: Detailed phylogeny of the mixed-family clade. CTLs identified in this study are indicated in red font. The orange in the structure represents the functional domain. Taxonomic families are distinguished by color blocks: Viperidae (yellow), mixed family (gray).

The basic form of lectin toxins is a single chain that can assemble into non-covalently linked complexes. In the Viperidae, a specialized class of lectin, often termed snaclecs or c-type lectins, exists as disulfide-linked dimers of two distinct subunits [36,37]. These lectins exhibit diverse thrombotoxic effects, including the inhibition of coagulation factors and thrombin. A total of 12 C-type lectin (CTL) sequences were identified (Figure 9). Of these, CTL10, CTL12, and CTL7 contained the EPN (glutamic acid + proline + asparagine) functional motif. This EPN-type CTL is conserved across many rear-fanged snakes and is considered as the ancestral form of lectin toxins. CTL4–CTL6, CTL2, and CTL11 were identified with QPD (glutamine + proline + aspartic acid) motif, which is thought to have arisen through convergent evolution. Conserved WIG and WXD motifs were present in all CTLs. Quantitative analysis revealed that CTL1 was the most abundant transcript among all identified CTLs. Notably, CTL1 lacked the canonical functional motifs (EPN or QPD), displaying characteristics typical of α-chains, including a QC (glutamine–cysteine) motif. This structural feature enables recognition of β-chains and promotes the assembly of snake C-type lectin-like proteins (snaclecs), which can exert strong prothrombotic activity or inhibit platelet aggregation [38]. Additionally, CTL7 and CTL8 exhibited cysteine patterns consistent with those of β-subunits. The phylogenetic tree comprises 211 sequences, including representatives from families beyond Viperidae, in which CTLs are commonly found. Unlike SVMPs and CRISPs, the CTL sequences in *R. lateralis* do not cluster within a conserved lineage. Instead, they are grouped into a large, mixed clade containing sequences from multiple families (Figure 10). This pattern likely reflects convergent evolution of this toxin class among various venomous snakes.

**Figure 11:**
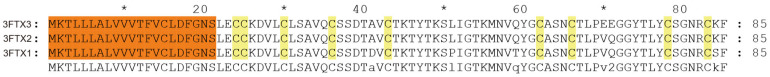
Multiple sequence alignment of three-finger toxins (3FTx). Signal peptides are highlighted in orange, and conserved cysteine residues are shaded in yellow. The asterisk denotes every 10 amino acids.

**Figure 12:**
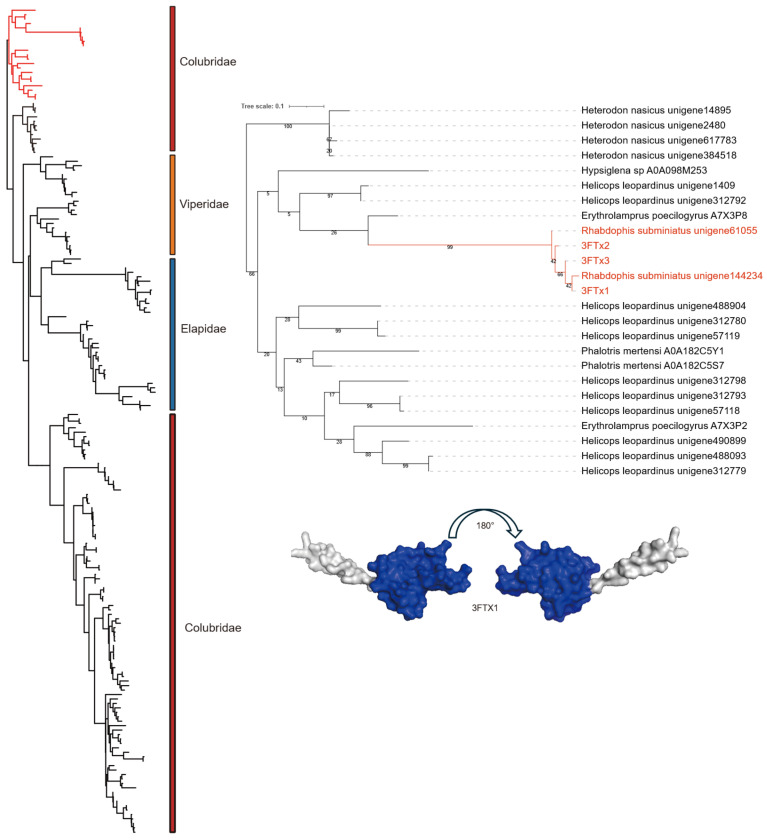
Phylogenetic analysis of three-finger toxins (3Ftxs). **Left**: The overall topology of the phylogenetic tree. The red branch indicates the clade expanded in the right panel. **Right**: Detailed phylogeny of the Colubridae lineage. *R. subminiatus*, which belongs to the same group as *R. lateralis*, is displayed in red font. In the three-dimensional protein model, blue represents functional domains. Taxonomic families are distinguished by color blocks: Viperidae (yellow), Elapidae (blue), and Colubridae (red).

Three-finger toxins (3FTxs), a family of non-enzymatic proteins, have been widely reported in snakes, particularly within the families Elapidae and Colubridae [39,40,41]. These toxins typically function as antagonists by binding to post-synaptic nicotinic acetylcholine receptors (nAChRs) [42], thereby inducing paralysis. However, because these toxins generally show low toxicity to mammals, snakes that predominantly express them, usually rear-fanged species specializing in avian or saurian prey, have often been regarded as non-venomous [43]. Previous studies have shown that three-finger toxins (3FTxs) evolved from non-secreted LY6 proteins through domain loss, thereby acquiring secretory capability [44]. In *R. lateralis*, the identified 3FTxs possess conserved signal peptide sequences and display the canonical arrangement of eight cysteine residues (Figure 11). These form four disulfide bonds, ensuring structural stability within the characteristic ‘finger’ loop regions. Transcriptomic analysis revealed that 3FTxs account for approximately 5% of total toxin expression across the three samples; they are not the predominant toxins in this species but may function as auxiliary components in envenomation. Phylogenetic analysis revealed that *R. lateralis* 3FTx sequences clustered within the Colubridae clade (Figure 12), showing a close relationship to those of the red-necked keelback (*R. subminiatus*), forming a distinct lineage characterized by long branch lengths.

## 3. Conclusions

Transcriptomic analysis of the *R. lateralis* Duvernoy’s gland revealed diverse toxin transcripts. Coagulation-related toxins dominated the expression profile, mainly P-III snake venom metalloproteinases (SVMPs) and C-type lectins (CTLs), whereas neurotoxic components, including cysteine-rich secretory proteins (CRISPs) and three-finger toxins (3FTxs), were present as secondary components. Historically, *R. lateralis* was regarded as non-venomous and medically insignificant. However, the predominant expression of SVMPs and CTLs likely underlies the severe coagulopathy associated with its envenomation, as these toxins disrupt the coagulation system in a manner consistent with the observed clinical symptoms [45]. Severe envenomation in *Rhabdophis* is confined to this species and *R. subminiatus*, the only two venomous species in the genus. This distinct pattern suggests a unique evolutionary trajectory. Phylogenetically, major toxins appear conserved within Colubridae. Moreover, despite clustering with related taxa, SVMPs, CRISPs, and 3FTxs exhibit substantial divergence, as reflected by their long branch lengths. In conclusion, although this species possesses potent coagulotoxic venom, the evolutionary drivers of this restricted toxicity within the genus require further investigation. Ultimately, these results expand the *Rhabdophis* toxin database, provide insights into rear-fanged snake venom evolution, and facilitate future research on clinical envenomation mechanisms.

## 4. Materials and Methods

### 4.1. Sample Collection and Transcriptome Sequencing

Two adult *R. lateralis* were collected from Xianning City, Hubei Province, and one adult from Guangyuan City, Sichuan Province, China. Following a one-month acclimatization period, both left and right Duvernoy glands were harvested and stored at −80 °C for RNA sequencing. Sequencing libraries were prepared using the NEBNext Ultra Directional RNA Library Prep Kit for Illumina (New England Biolabs, Ipswich, MA, USA), following the manufacturer’s protocol. Briefly, mRNA was purified from total RNA using probes to remove rRNA. Fragmentation was carried out using divalent cations under elevated temperature in First Strand Synthesis Reaction Buffer (5X). The first strand cDNA was synthesized using random hexamer primer and M-MuLV Reverse Transcriptase. Second strand cDNA synthesis was subsequently performed using DNA Polymerase I (Thermo Fisher Scientific, Waltham, MA, USA) and RNase H, with remaining overhangs converted into blunt ends via exonuclease and polymerase activities. After adenylation of 3′ ends of DNA fragments, adaptors with a hairpin loop structure were ligated to prepare for hybridization. To select cDNA fragments of 370–420 bp in length, the library fragments were purified with AMPure XP system (Beckman Coulter, Brea, CA, USA). Then, 3 µL of USER Enzyme was used with size-selected, adaptor-ligated cDNA and incubated at 37 °C for 15 min, followed by inactivation at 95 °C for 5 min. PCR amplification was performed using Phusion High-Fidelity DNA polymerase (Thermo Fisher Scientific, Waltham, MA, USA) to enrich the cDNA library; PCR products were purified using the AMPure XP system and library quality was assessed with the Agilent 5400 system (Agilent Technologies, Santa Clara, CA, USA) and quantification via PCR (1.5 nM). Qualified libraries were sequenced on Illumina platforms (Illumina, San Diego, CA, USA) with PE150 strategy in Smartgenomics Technology Institute (Tianjin, China) according to effective library concentration and data amount required.

### 4.2. Transcriptome Assembly and Annotation

We trimmed Illumina adapters and removed low-quality reads using Fastp v0.23.4 [46]. Paired-end reads were merged using Pear v0.9.6 [47]. To assemble a high-quality transcriptome de novo, we used four distinct assemblers: trinity v2.15.1 [48] with default parameters, extender [49], spades-trans v3.15.5 [50] and SOAPdenovo-Trans v 1.03 with the 31-mer and 127-mer option [51]. We combined the assembly results and used cd-hit-est v4.8.1 [52] to reduce redundancy. We used ToxCodan v1.0 [53] to annotate toxin and non-toxin transcripts. The process involved: (i) similarity-based alignment of coding sequences against ToxCodan’s curated toxin repository, (ii) integration with SignalP v4.0-predicted secretory peptides. The toxin CDS was then classified into toxin families and CD-HIT v4.8.1 was used to cluster the sequences within each family at 99% sequence similarity.

Coding sequences of non-toxin transcripts were predicted using CodAn v1.0 [54] with the vertebrates model and subsequently searched against the ToxCodan protein database and SwissProt (accessed on 22 May 2025) using BLAST v2.16.0 [55]. Unannotated CDSs were searched against vertebrate BUSCO [56] models and the Pfam (https://pfam.xfam.org/, accessed on 24 May 2025) database using HMMER v3.2.1 [57]. Predicted CDSs were translated into protein sequences and functionally annotated by alignment against public databases (accessed on 24 May 2025). Following annotation, chimeric sequences were removed using ChimeraKiller v0.74 (https://github.com/masonaj157/ChimeraKiller, accessed on 27 May 2025). Transcripts from each individual were clustered using cd-hit-est at 98% identity. Subsequently, these were merged and re-clustered at the same threshold to generate a consensus transcriptome for the three *R. lateralis* samples.

### 4.3. Expression Analyses and Ortholog Identification

Transcript quantification was performed using Bowtie2 v2.5.4 [58] and RSEM v1.3.3 [59], with TPM (transcripts per million) as the abundance metric. Non-toxin transcripts with TPM > 1 were retained. The proportional expression of each toxin family was calculated by ‘dplyr’ and visualized in RStudio v2025.09.1 using ‘ggplot2’. We also explored the phylogenetic relationships of SVMP, CRISP, CTL, and 3FTX toxins compared to other snakes. The coding sequences (CDSs) were translated using Transeq [60], combined with the collected dataset, aligned using MUSCLE v5.3 [61] and trimmed; all aligned sequences are provided in the Supplementary Files (Supplementary File S1). Poorly aligned regions were retained. Maximum likelihood trees were constructed using RAxML v1.2.0 [62] under the JTT substitution model with 1000 bootstraps. Visualization of multiple sequence alignments was performed using GENEDOC [63]. The four toxin protein structures were predicted using AlphaFold3 [64]. The model with the highest pLDDT (predicted Local Distance Difference Test) score was selected for each toxin.

## Figures and Tables

**Table 1:** Summary statistics of the *R. lateralis* Duvernoy’s gland transcriptome.

Sample	Raw_Bases	Clean_Bases	Clean_Reads	Q20 Rate (%)	Q30 Rate (%)	GC_Count (%)
*R. lateralis*_A	7,245,753,600	7,182,157,778	48,304,610	99.38	97.64	45.62
*R. lateralis*_B	7,554,168,300	7,514,922,486	50,360,694	99.37	97.65	44.77
*R. lateralis*_C	11,377,664,400	11,332,613,482	75,841,036	99.20	97.07	45.18

**Table 2:** Number of sequences after assembly and processing.

Sample	Contigs	CDS Count	Final Sequences (Post-Chimera)
A	226,498	17,000	4552
B	250,150	14,970	3040
C	532,251	19,291	4420

## Data Availability

The raw sequence data generated in this paper have been deposited in the Genome Sequence Archive in National Genomics Data Center, China National Center for Bioinformation, Chinese Academy of Sciences (GSA: CRA039926), that are publicly accessible at https://ngdc.cncb.ac.cn/gsa (accessed on 9 February 2026). The cds sequence of toxins has been uploaded to figshare (https://doi.org/10.6084/m9.figshare.31742212).

## Supplementary Materials

The following supporting information can be downloaded at: https://www.mdpi.com/article/10.3390/toxins18040167/s1, Supplementary File S1: Aligned sequences used for the phylogenetic tree.
